# Longitudinal changes of inflammatory parameters and their correlation with disease severity and outcomes in patients with COVID-19 from Wuhan, China

**DOI:** 10.1186/s13054-020-03255-0

**Published:** 2020-08-27

**Authors:** Zhilin Zeng, Haijing Yu, Huilong Chen, Weipeng Qi, Liang Chen, Guang Chen, Weiming Yan, Tao Chen, Qin Ning, Meifang Han, Di Wu

**Affiliations:** Department and Institute of Infectious Disease, Tongji Hospital, Tongji Medical College, Huazhong University of Science and Technology, No. 1095, Jiefang Avenue, Wuhan, 430030 China

**Keywords:** COVID-19, SARS-CoV-2, Inflammatory mediators, Cytokine, Disease severity, Outcome

## Abstract

**Background:**

Coronavirus disease 2019 (COVID-19) is a newly emerging infectious disease and rapidly escalating epidemic caused by the severe acute respiratory syndrome coronavirus 2 (SARS-CoV-2). The pathogenesis of COVID-19 remains to be elucidated. We aimed to clarify correlation of systemic inflammation with disease severity and outcomes in COVID-19 patients.

**Methods:**

In this retrospective study, baseline characteristics, laboratory findings, and treatments were compared among 317 laboratory-confirmed COVID-19 patients with moderate, severe, or critically ill form of the disease. Moreover, the longitudinal changes of serum cytokines, lactate dehydrogenase (LDH), high-sensitivity C-reactive protein (hsCRP), and hsCRP to lymphocyte count ratio (hsCRP/L) as well as their associations with disease severity and outcomes were investigated in 68 COVID-19 patients.

**Results:**

Within 24 h of admission, the critically ill patients showed higher concentrations of inflammatory markers including serum soluble interleukin (IL)-2 receptor, IL-6, IL-8, IL-10, tumor necrosis factor alpha (TNF-α), ferritin, procalcitonin, LDH, hsCRP, and hsCRP/L than patients with severe or moderate disease. The severe cases displayed the similar response patterns when compared with moderate cases. The longitudinal assays showed the levels of pro-inflammatory cytokines, LDH, hsCRP, and hsCRP/L gradually declined within 10 days post admission in moderate, severe cases or those who survived. However, there was no significant reduction in cytokines, LDH, hsCRP, and hsCRP/L levels in critically ill or deceased patients throughout the course of illness. Compared with female patients, male cases showed higher serum concentrations of soluble IL-2R, IL-6, ferritin, procalcitonin, LDH, and hsCRP. Multivariate logistic regression analysis revealed that IL-6 > 50 pg/mL and LDH > 400 U/L on admission were independently associated with disease severity in patients with COVID-19.

**Conclusion:**

Exuberant inflammatory responses within 24 h of admission in patients with COVID-19 may correlate with disease severity. SARS-CoV-2 infection appears to elicit a sex-based differential immune response. IL-6 and LDH were independent predictive parameters for assessing the severity of COVID-19. An early decline of these inflammation markers may be associated with better outcomes.

## Introduction

Two coronaviruses, including severe acute respiratory syndrome (SARS) and Middle East respiratory syndrome (MERS) have been known to cause fatal pneumonia outbreak in the past two decades [1, 2]. In December 2019, a cluster of pneumonia cases of unknown origin emerged in Wuhan, China [3], which exhibits a considerable phylogenetic similarity to severe acute respiratory syndrome coronavirus (SARS-CoV) [4]. Subsequently, the virus and associated disease had been formally named severe acute respiratory syndrome coronavirus 2 (SARS-CoV-2) and coronavirus infection disease-19 (COVID-19), retrospectively [4]. The World Health Organization has declared COVID-19 is pandemic and constituted a public health emergency of international concern. As of July 9, 2020, a total of 11,841,326 laboratory-confirmed cases and a mortality of approximately 4.6% had been documented globally, posing unprecedented challenges to global public health [5].

Patients infected with SARS-CoV-2 present with a wide range of clinical severity varying from asymptomatic to fatal condition [6, 7]. Advanced age and underlying comorbidities are risk factors for higher severity of illness and death from COVID-19 [3, 8–10]. Disturbance of the immune system in patients has been considered as one of the hallmarks for COVID-19, especially cytokine release syndrome and lymphopenia [11, 12]. The autopsy study of COVID-19 pneumonia implied that overactivation of T cells, manifested by increase of Th17 and high cytotoxicity of CD8^+^ T cells, accounts for, at least in part, the severe immune injury in COVID-19 patients [13]. Evidence has proven that COVID-19-related lung injury and extra-pulmonary organ dysfunction include acute respiratory distress syndrome (ARDS) like presentation, cardiac injury, kidney injury, liver injury, and sepsis as well as coagulation disorders [3, 8–10, 14, 15]. Those results gave credence to the view that SARS-CoV-2 infection was not only a pulmonary disease but also a systemic inflammatory illness. However, the mechanisms underlying pathogenesis of the pulmonary and extrapulmonary injury of COVID-19 remain poorly defined.

As a double-edged sword, the activation of immune systems plays a pivotal role in protecting against infectious agents; in the meantime, it is accompanied by inflammatory mediator release. High inflammatory cytokines levels have been strongly correlated with poor disease outcomes in respiratory virus infection [16]. Evidence has proven that massive inflammatory cell infiltration and marked pro-inflammatory cytokine responses induced by SARS-CoV and MERS-CoV infection played a crucial role in disease progression [17, 18]. The information on mechanisms by which SARS-CoV-2 caused severe illness and lethal outcomes is limited.

Recently, our preliminary study reported that levels of inflammatory mediators were significantly higher in severe cases compared with non-severe cases of COVID-19 [19, 20]. Huang et al. found that intensive care unit (ICU) patients had higher serum levels of interleukin (IL)-10, tumor necrosis factor alpha (TNF-α), procalcitonin (PCT), and lactate dehydrogenase (LDH) compared with non-ICU patients [3]. Zhou et al. reported that levels of plasma ferritin (Fer), LDH, and IL-6 were markedly elevated in deceased patients than in survivors [8]. Taken together, these findings suggested hyperactive immune responses mainly manifesting as increased inflammatory markers could be associated with COVID-19 disease severity and outcomes. However, the longitudinal changes of inflammatory parameters throughout disease progression of COVID-19 and their correlation with disease severity and outcomes warrant further investigation.

In order to enrich the knowledge about the immunopathology of SARS-CoV-2 infection, we characterized the changes of serum inflammatory mediators in the COVID-19 patients with different disease severity and outcomes in this retrospective case series. Comparative and longitudinal analyses may unveil the association of inflammatory parameters with disease severity and outcomes of COVID-19.

## Methods

### Study design and participants

We conducted a retrospective study focusing on the adult hospitalized patients with COVID-19 from Tongji Hospital, Tongji Medical College, Huazhong University of Science and Technology from January 28, 2020, to February 12, 2020. The Tongji Hospital, located in Wuhan, is the largest medical center for patients with moderate, severe, or critically ill form of COVID-19 designated by local authority. This study was approved by the Ethical Committee of Tongji Hospital. Data were anonymous and the requirement for informed consent was waived owing to the rapid emergence of this infectious disease.

Oropharyngeal swab specimens were collected for extracting COVID-19 RNA from patients. All patients with SARS-CoV-2 were confirmed using quantitative real-time reverse transcription polymerase chain reaction (RT-PCR) assay. Three hundred seventeen patients who had available data on inflammatory parameters within 24 h of admission were enrolled in this retrospective study. Among them, 68 patients with variable disease severity who had longitudinal data available on cytokines, LDH, high-sensitivity C-reactive protein (hsCRP), and hsCRP to lymphocyte count ratio (hsCRP/L) were included in the further analysis.

### Data collection

Medical record information including clinical, laboratory, and treatment as well as outcome data were extracted by using data collection forms. The data collection forms were checked independently by two trained physicians.

### Definition

All of the included patients were diagnosed with COVID-19 according to the Guidance for Corona Virus Disease 2019 (6th edition) released by the National Health Commission of China [21]. According to this guidance, patients were classified as follows: (1) mild cases: the clinical symptoms are mild and no pneumonia manifestation can be found in imaging; (2) moderate cases: patients have symptoms like fever and respiratory tract symptoms, etc., and pneumonia manifestation can be seen in imaging; (3) severe cases: patients meet any of the following: (i) respiratory distress, respiratory rates ≥ 30 breaths/minute; (ii) the oxygen saturation ≤ 93% at a rest state; (iii) arterial oxygen tension (PaO_2_) over inspiratory oxygen fraction (FIO_2_) ratio ≤ 300 mmHg (1 mmHg = 0.133 kPa); and (iv) multiple pulmonary lobes showing more than 50% progression of lesion in 24–48 h on imaging; and (4) critically ill cases: patients meet any of the following: (i) respiratory failure occurs and mechanical ventilation is required; (ii) shock occurs; (iii) complicated with other organ failure that requires monitoring and treatment in the ICU.

The endpoint was the in-hospital death. The clinical data including inflammatory parameters and outcomes were monitored up to March 13, 2020, the final date of follow-up.

### Principles of management of patients

Vital signs and oxygen saturation should be monitored (patients with severe disease need continuous monitoring), supportive treatment strengthened, sufficient calories provided, and the stability of the internal environment, such as water, electrolyte, and acid-base balance, maintained.

Supplemental oxygen therapy should be given immediately to patients with hypoxemia. The target oxygen saturation is pulse oxygen saturation ≥ 90% in patients. If standard oxygen therapy fails, high-flow nasal catheter oxygen or non-invasive ventilation can be used. If no improvement is seen of non-invasive mechanical ventilation, invasive mechanical ventilation should be used.

As no therapy was proved effectively, anti-virus (oseltamivir and arbidol) was empirically administered. Antibiotics (oral and intravenous) and corticosteroid therapy were given by experienced physicians according to patient’s condition.

### Inflammatory parameter measurements

Inflammatory indicators were conducted in the Department of Clinical Laboratory in Tongji Hospital.

Blood samples were processed according to hospital’s standard procedures, including a blood withdrawn into a vacutainer tube containing coagulant for serum collection. The samples were centrifuged for 10 min at 2000*g*. Serum was then collected and tested within 4–6 h. All procedures were performed under level 3 protection. Cytokines including interleukin-2 receptor (sIL-2R), IL-6, IL-8, IL-10, and TNF-α were assessed in serum samples drawn shortly at each time points by chemiluminescence immunoassay (CLIA) performed on a fully automated analyzer (Immulite 1000, DiaSorin Liaison, Italy or Cobas e602, Roche Diagnostics, Germany) for all patients according to the manufacturer’s instructions. IL-2R kit (#LKIP1), IL-8 kit (#LK8P1), IL-10 kit (#LKXP1), and TNF-α kit (#LKNF1) were purchased from DiaSorin (Vercelli, Italy). IL-6 kit (#05109442 190) was purchased from Roche Diagnostics, Germany. HsCRP was detected by immunoturbidimetry method according to Nippon Denkasei Co., Ltd. instruction. PCT and Fer were tested by Roche electrochemiluminescence and granule-enhanced immunoturbidimetry method respectively. The following normal range values were used in the present study: sIL-2R 5 U/mL (223–710 U/mL), IL-6 1.5 pg/mL (0–7.0 pg/mL), IL-8 5 pg/mL (0–62 pg/mL), IL-10 5 pg/mL (0–9.1 pg/mL), TNF-α 4 pg/mL (0–8.1 pg/mL), hsCRP 0.1 mg/L (0–1 mg/L), PCT 0.02 ng/mL (0.02–0.05 ng/mL), Fer 5 μg/L (Male 30–400 μg/L, Female 15–150 μg/L), and LDH 10 U/L (0–250 U/L).

### Statistical analysis

We summarized continuous variables as medians with interquartile ranges (IQR) or mean ± standard deviation unless otherwise indicated. Shapiro-Wilk test was conducted to assess whether continuous variables follow normal distribution. Levene’s test was used to analyze the homogeneity of variance. Age, hemoglobin, albumin and blood bicarbonate ions were normally distributed and homogeneous variables, but the other variables were not. ANOVA analysis and Student’s *t* test were performed in normally distributed and homogeneous data among the three groups with different disease severities as well as between survivors and non-survivors respectively. Otherwise, Kruskal-Wallis test and the Mann-Whitney-Wilcoxon test were applied where appropriate. One-way ANOVA with repeated measures were performed in longitudinal variables with normal distribution and post-hoc analysis with Bonferroni correction was used when significant differences were observed. Friedman test with a post hoc option was used to analyze longitudinal data with abnormal distribution. Categorical variables were expressed as percentages and compared by chi-square test or Fisher exact test. Univariate logistic regression and multivariate logistic regression were performed to investigate association of independently variables with disease severity. A two-sided *α* of less than 0.05 was considered statistically significant. Statistical analyses were done with SPSS software (version 22.0.) and GraphPad Prism 6.

## Results

### Clinical characteristics, baseline laboratory findings, and treatments of COVID-19 patients

Three hundred seventeen adult patients were enrolled in this retrospective study, and serum inflammatory parameters levels were measured within 24 h of admission. Ninety-three patients were classified as moderate, 167 as severe, and 57 as critically ill. As of March 13, 2020, 40 (12.6%) patients eventually died of COVID-19. As shown in Table 1 and Table S1 (see Additional file 1), of these 317 patients, the median age was 62.0 years (IQR 51.0–70.0), with approximately equal numbers of males (51.1%) and females. More than half of the patients had underlying comorbidities, with hypertension (39.1%) being the most common comorbidity followed by diabetes (19.9%). The most common symptoms at disease onset were fever (89.6%) and cough (75.7%), followed by shortness of breath (47.3%). The most frequent laboratory abnormalities noted were lymphopenia, hypoalbuminemia, thrombocytopenia, elevated blood bicarbonate ions, increased urea nitrogen, and creatinine in critically ill patients.

**Table 1:** Clinical characteristics and baseline laboratory findings of patients infected with SARS-CoV-2

	All patients (*n* = 317)	Moderate patients (*n* = 93)	Severe patients (*n* = 167)	Critical patients (*n* = 57)
**Characteristics**				
Age, years	62.0 (51.0–70.0)	59.0 (46.0–68.5)	62.0 (51.0–69.0)*	68.0 (57.0–77.0)*
Males, *n* (%)	162 (51.1%)	41 (44.1%)	90 (53.9%)	31 (54.4%)
Comorbidity				
Chronic respiratory diseases, *n* (%)	19 (6.0%)	4 (4.3%)	9 (5.9%)	6 (10.5%)
Hypertension, *n* (%)	124 (39.1%)	40 (43.0%)	61 (36.5%)	23 (40.4%)
Coronary artery disease, *n* (%)	30 (9.5%)	10 (10.8%)	14 (8.4%)	6 (10.5%)
Diabetes mellitus, *n* (%)	63 (19.9%)	17 (18.3%)	30 (18.0%)	16 (28.1%)
Chronic kidney disease, *n* (%)	4 (1.3%)	3 (3.2%)	1 (0.6%)	0
Tumor, *n* (%)	6 (1.9%)	3 (3.2%)	2 (1.2%)	1 (1.8%)
**Laboratory findings**				
White blood cell count, × 10^^^9/L	5.6 (4.4–7.8)	5.4 (4.2–6.7)	5.2 (4.2–7.2)	8.6 (5.9–13.4)*^#^
Neutrophil count, × 10^^^9/L	4.0 (2.9–6.3)	3.5 (2.6–4.3)	3.9 (2.8–5.4)	7.5 (4.7–12.4)*^#^
Lymphocyte count, × 10^^^9/L	0.9 (0.6–1.3)	1.0 (0.8–1.5)	0.9 (0.7–1.2)	0.6 (0.5–0.8)*
Hemoglobin, g/L	127.0 (116.0–139.0)	125.0 (114.5–134.0)	127.0 (116.0–139.0)	133.0 (117.5–140.5)
Platelet count, × 10^^^9/L	208.0 (154.0–285.0)	223.0 (173.0–307.5)	201.0 (151.0–276.0)	181.0 (124.0–257.0)*
Albumin, g/L	33.7 (30.8–36.8)	36.4 (32.9–40.0)	33.5 (31.2–36.2)*	30.7 (28.2–33.4)*^#^
Blood bicarbonate ions, mmol/L	23.5 (21.7–25.1)	23.9 (21.9–25.6)	23.6 (22.0–25.0)	22.2 (19.3–24.3)*^#^
Blood urea nitrogen, mmol/L	4.6 (3.5–6.0)	4.0 (3.3–5.0)	4.4 (3.5–5.5)	7.2 (5.1–10.7)*^#^
Blood creatinine, μmol/L	70.0 (57.5–86.0)	62.0 (55.0–81.5)	69.0 (59.0–84.0)	79.0 (62.0–103.5)*^#^
Lactate dehydrogenase, U/L	302.0 (237.0–425.0)	234.0 (209.0–283.5)	307.0 (249.0–392.0)*	496.0 (415.0–690.0)*^#^
High-sensitivity C-reactive protein, mg/L	41.1 (11.8–90.6)	14.0 (4.8–39.7)	44.1 (15.4–89.0)*	93.0 (65.0–165.1)*^#^
Procalcitonin, ng/mL	0.06 (0.03–0.17)	0.04 (0.02–0.07)	0.06 (0.03–0.14)*	0.21 (0.10–0.70)*^#^
Ferritin, μg/L	751.5 (435.7–1333.9)	504.0 (282.0–776.4)	784.0 (456.1–1325.6)*	1340.0 (884.6–1989.4)*^#^
Soluble interleukin-2 receptor, U/mL	762.0 (509.9–1124.0)	655.0 (483.5–916.5)	762.0 (576.0–1060.0)*	1174.0 (915.0–1552.5)*^#^
Interleukin-6, pg/mL	21.7 (7.3–53.9)	13.1 (3.8–23.5)	21.7 (6.3–53.9)*	59.7 (33.5–137.4)*^#^
Interleukin-8, pg/mL	15.5 (9.4–26.6)	12.2 (7.8–9.0)	15.5 (9.7–26.1)*	26.0 (14.9–49.4)*^#^
Interleukin-10, pg/mL	5.6 (5.00–9.4)	5.0 (5.0–6.8)	5.3 (5.0–8.8)	9.5 (6.7–15.9)*^#^
Tumor necrosis factor alpha, pg/mL	9.1 (7.2–12.1)	8.7 (6.8–10.8)	9.0 (7.1–11.6)*	11.0 (8.0–14.8)*^#^
hsCRP/lymphocyte, × 10^^^-9 mg	53.5 (10.5–128.1)	10.8 (3.4–49.6)	56.0 (15.8–115.4)*	142.1 (82.3–300.4)*^#^

Data are expressed as median (IQR) or *n* (%). *P < 0.05 represents significant differences between severe or critically ill group and moderate group, ^#^*P* < 0.05 represents significant differences between critically ill group versus severe group

With regard to treatment during course of hospitalization, systematic corticosteroid use differed significantly among the three groups. Oxygen support was most frequently used in the critically ill patients. There were significant differences in terms with age, prevalence of chest tightness, and respiratory rate between critically ill and moderate patients. Nearly all laboratory findings (except hemoglobin) and treatment options (except antivirals) differed markedly between critically ill and moderate patients. Difference in prevalence of chest tightness, laboratory findings (including white blood cell count, neutrophil count, albumin, blood bicarbonate ions, and blood creatinine) and treatment (except antivirals) were noted between severe patients and critically ill patients (Table 1 and Table S1 in Additional file 1).

Clinical characteristics, baseline laboratory findings, and treatment of patients with available data on serial cytokines, LDH, hsCRP, and hsCRP/L measurement were shown in Table 2 and Table S2 (see Additional file 1).

**Table 2:** Baseline laboratory findings of survivors and deceased patients with serial changes of inflammatory parameters

	All patients (*n* = 68)	Survivors (*n* = 54)	Deceased patients (*n* = 14)
White blood cell count, × 10^^^9/L	6.5 (4.5–9.3)	5.6 (4.3–9.3)	7.2 (6.0–11.1)
Neutrophil count, × 10^^^9/L	4.9 (3.1–7.8)	4.2 (2.7–7.2)	6.5 (5.0–9.6)
Lymphocyte count, × 10^^^9/L	0.8 (0.7–1.1)	0.9 (0.7–1.1)	0.7 (0.5–0.9)*
Hemoglobin, g/L	130.5 (117.5–141.8)	125.0 (116.0–141.3)	138.5 (135.3–145.8)*
Platelet count, × 10^^^9/L	229.5 (168.8–293.3)	236.5 (166.0–299.3)	223.5 (193.5–260.3)
Albumin, g/L	32.0 (29.2–34.0)	32.3 (29.3–34.3)	31.2 (28.5–33.7)
Blood bicarbonate ions, mmol/L	23.6 (22.1–24.8)	23.8 (22.4–24.8)	22.3 (19.8–24.4)
Blood urea nitrogen, mmol/L	4.9 (3.6–7.1)	4.6 (3.6–6.4)	7.7 (5.7–10.2)*
Blood creatinine, μmol/L	71.0 (61.5–87.0)	70.5 (59.5–85.0)	78.5 (62.5–96.0)
hsCRP/lymphocyte, × 10^^^9 mg	94.4 (45.9–184.1)	86.5 (38.5–144.8)	140.4 (79.4–330.6)*

Data are expressed as median (IQR) or *n* (%). *P < 0.05 compared with the survivors

### Levels of inflammatory parameters in the serum among moderate, severe, and critically ill patients

The serum concentrations of common inflammatory makers and cytokines, including sIL-2R, IL-6, IL-8, IL-10, TNF-α, PCT, Fer, LDH, hsCRP, and hsCRP/L were measured in moderate, severe, and critically ill patients. The levels of inflammatory parameters were elevated in the serum of critically ill patients compared with moderate and severe cases (Table 1). When compared to severe patients, moderate patients also showed lower levels of inflammatory makers, except for IL-10 (Table 1). These findings indicated that elevated levels of inflammatory markers were associated with disease severity.

### Longitudinal changes of inflammatory parameters in COVID-19 patients with different disease severity

**Fig. 1:**
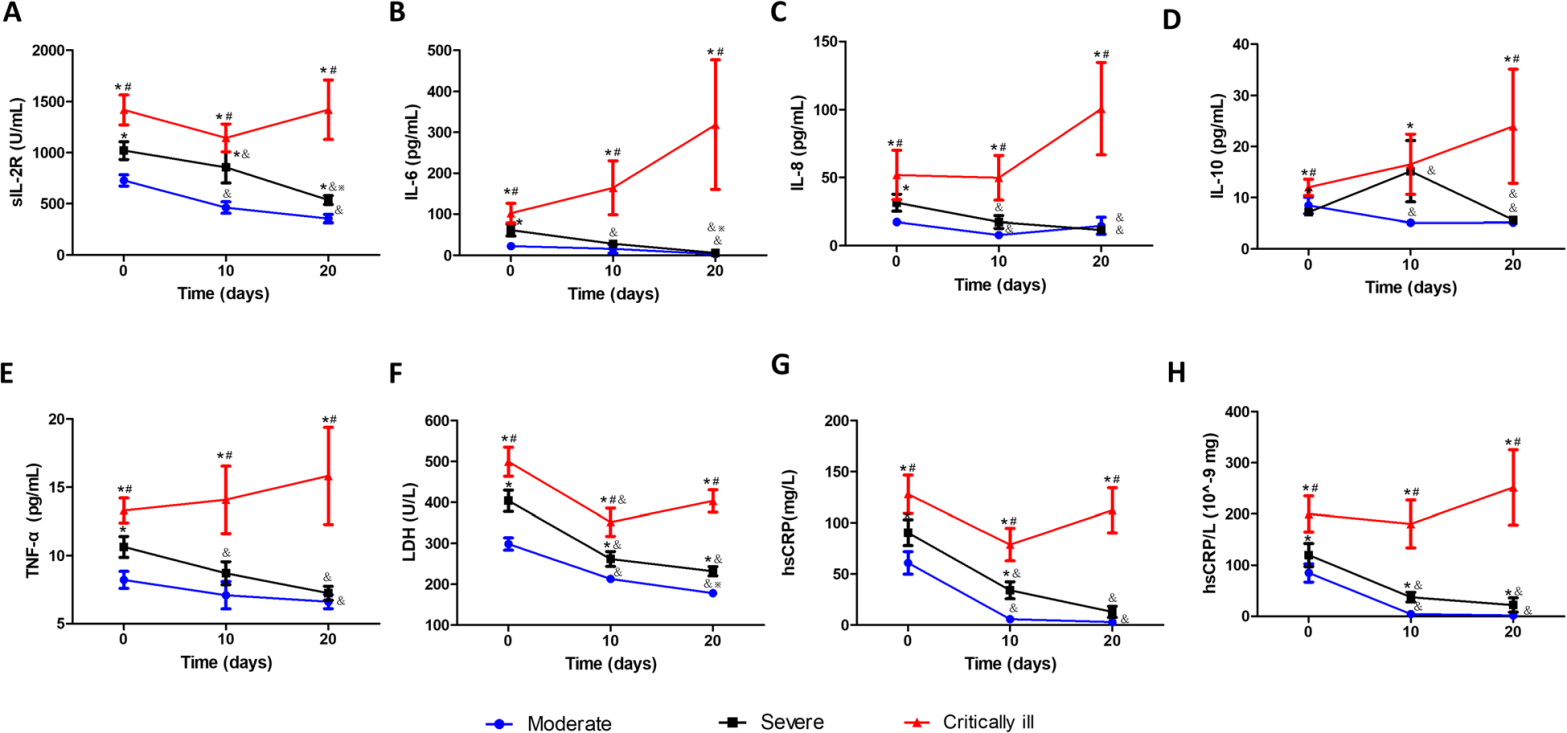
Longitudinal changes of inflammatory parameters in COVID-19 patients with different disease severity. The levels of sIL-2R (**a**), IL-6 (**b**), IL-8 (**c**), IL-10 (**d**), TNF-α (**e**), LDH (**f**), hsCRP (**g**), and hsCRP/L(**h**) in moderate (blue circle, *N* = 18), severe (black square, *N* = 29), and critically ill (red triangle, *N* = 21) patients were determined at various time points. Data are expressed as mean ± SEM. *P < 0.05 indicates difference between severe or critically ill patients versus the moderate patients, ^#^*P* < 0.05 indicates difference between critically ill patients versus the severe patients, ^&^*P* < 0.05 indicates difference between day 10 or 20 versus day 0, and ^※^*P* < 0.05 indicates difference between day 20 versus day 10

Subgroup patients with available data on longitudinal cytokines, LDH, hsCRP, and hsCRP/L measurement were analyzed to investigate their serial changes with respect to disease severity. Levels of cytokines, LDH, hsCRP, and hsCRP/L at three time points were collected and presented. The first time point (day 0) was their baseline concentrations within 24 h of admission. The third one (day 20) was the levels of these inflammatory parameters measured before discharge or death roughly on hospital day 20 (ranging from day 15–25). The second time point (day 10) was the levels of these inflammatory parameters roughly on hospital day 10 (ranging from day 5–15). Compared to both moderate and severe patients, critically ill patients showed significant increase in serum concentrations of sIL-2R, IL-6, IL-8, TNF-α, LDH, hsCRP, and hsCRP/L during the course of hospitalization. Both severe and critically ill patients showed markedly increased IL-10 levels on hospital day 10 as compared to moderate cases, whereas only critically ill patients had sustained high levels of IL-10 throughout the disease course (Fig. 1d). After receiving medical treatment, both moderate and severe patients showed a gradual decrease in pro-inflammatory cytokines, LDH, hsCRP, and hsCRP/L levels during hospitalization (Fig. 1). It is also intriguing to note that IL-10 concentrations elevated on day 10 in the beginning and then decreased to the minimum thereafter in severe patients (Fig. 1d). In contrast, there was an upward trend of these inflammatory makers in critically ill cases, despite no statistical difference (Fig. 1).

Laboratory findings in COVID-19 patients together with different disease severities at three time points were shown in Figure S1 (see Additional file 2).

### Longitudinal changes of inflammatory parameters in survivors and deceased patients

**Fig. 2:**
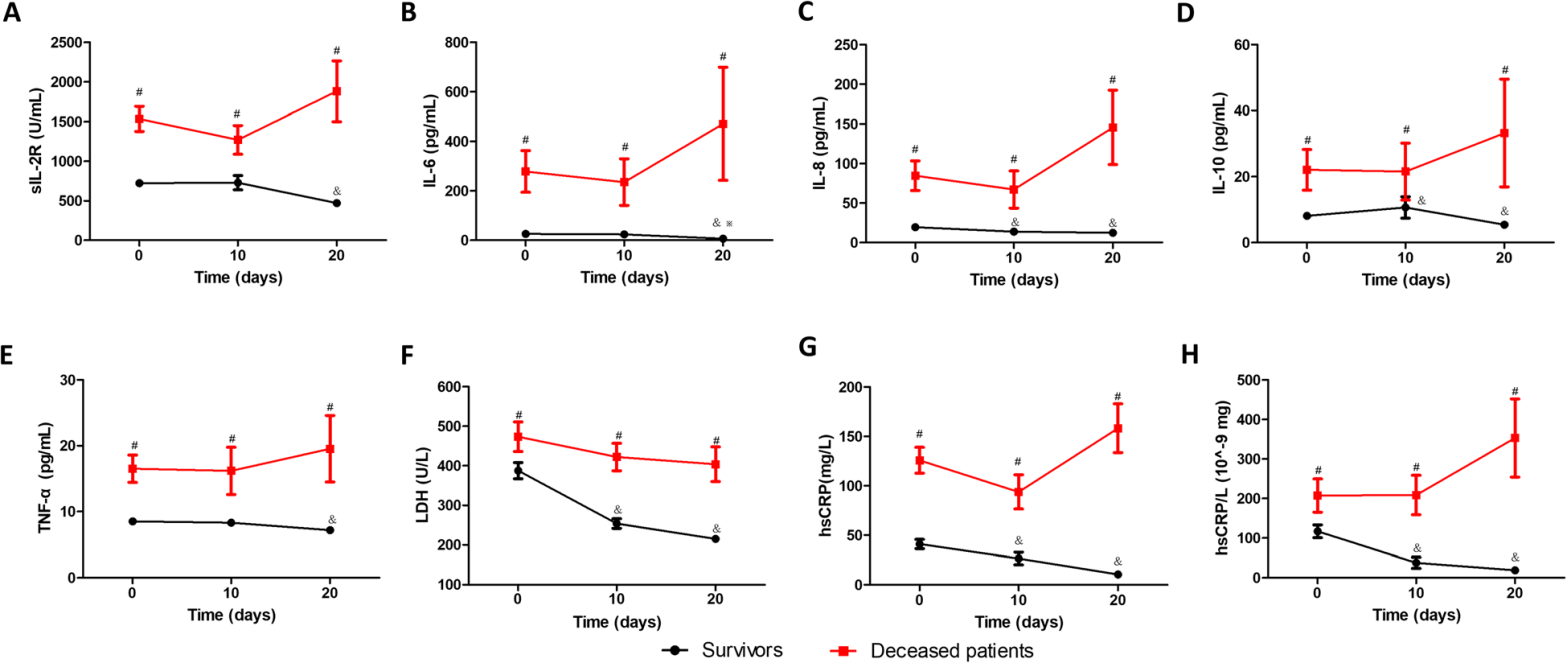
Longitudinal changes of inflammatory parameters in survivors and deceased patients. The levels of sIL-2R (**a**), IL-6 (**b**), IL-8 (**c**), IL-10 (**d**), TNF-α (**e**)), LDH (**f**), hsCRP (**g**), and hsCRP/L(**h**) in survivors (black circle, *N* = 54) and deceased patients (red square, *N* = 14) were determined at various time points. Data are expressed as mean ± SEM. ^#^*P* < 0.05 indicates difference between survivors and deceased patients, ^&^*P* < 0.05 indicates difference between day 10 or 20 versus day 0, and ^※^*P* < 0.05 indicates difference between day 20 versus day 10

Serum concentrations of cytokines, LDH, hsCRP, and hsCRP/L during hospitalization were significantly higher in the deceased patients than in those who recovered (Fig. 2). More importantly, these inflammatory parameters concentrations were sustained high without decrease in deceased patients during hospitalization (Fig. 2), whereas levels of pro-inflammatory cytokines, LDH, hsCRP, and hsCRP/L were gradually declined in survivors (Fig. 2). Intriguingly, the serum IL-10 levels in survivors increased on day 10 and then fell to lowest level on day 20 (Fig. 2d).

Comparisons of laboratory parameters between survivors and deceased patients at three time points were shown in Figure S2 (see Additional file 2).

### Comparison of inflammatory markers and laboratory parameters between male and female patients

As shown in Table 3, the concentrations of several inflammatory markers, including sIL-2R, IL-6, Fer, PCT, LDH, and hsCRP, were markedly higher in the serum of male patients compared with female cases. Similarly, male patients showed higher levels of white blood cell count, neutrophil count, hemoglobin, blood urea nitrogen, and blood creatinine. On the contrary, male patients exhibited lower levels of platelet count and albumin.

**Table 3:** Baseline laboratory findings of female and male patients infected with SARS-CoV-2

	All patients (*n* = 317)	Females (*n* = 155)	Males (*n* = 162)	*P* value
White blood cell count, × 10^^^9/L	5.6 (4.4–7.8)	5.2 (4.1–7.5)	5.8 (4.8–8.0)	0.045
Neutrophil count, × 10^^^9/L	4.0 (2.9–6.3)	3.6 (2.6–5.9)	4.3 (3.1–6.4)	0.024
Lymphocyte count, × 10^^^9/L	0.9 (0.6–1.3)	1.0 (0.7–1.3)	0.8 (0.6–1.1)	0.45
Hemoglobin, g/L	127.0 (116.0–139.0)	120.0 (112.0–128.0)	136.0 (125.8–144.0)	< 0.001
Platelet count, ×10^^^9/L	208.0 (154.0–285.0)	233.0 (164.0–307.0)	190.0 (141.8–254.0)	< 0.001
Albumin, g/L	33.7 (30.8–36.8)	34.0 (31.3–37.8)	33.4 (30.2–36.1)	0.037
Blood bicarbonate ions, mmol/L	23.5 (21.7–25.1)	23.8 (21.8–25.5)	23.3 (21.6–24.8)	0.231
Blood urea nitrogen, mmol/L	4.6 (3.5–6.0)	4.2 (3.2–5.3)	4.9 (3.8–6.5)	< 0.001
Blood creatinine, μmol/L	70.0 (57.5–86.0)	58.0 (52.0–70.0)	81.0 (68.0–95.0)	< 0.001
Lactate dehydrogenase, U/L	302.0 (237.0–425.0)	272.0 (223.0–345.0)	324.0 (254.0–471.5)	< 0.001
High-sensitivity C-reactive protein, mg/L	41.1 (11.8–90.6)	23.4 (7.1–69.3)	66.0 (25.9–121.4)	< 0.001
Procalcitonin, ng/mL	0.06 (0.03–0.17)	0.04 (0.02–0.09)	0.09 (0.05–0.23)	< 0.001
Ferritin, μg/L	751.5 (435.7–1333.9)	533.2 (264.8–932.6)	1069.85 (611.4–1734.2)	< 0.001
Soluble interlukin-2 receptor, U/mL	762.0 (509.9–1124.0)	678.0 (501.0–1023.0)	879.0 (625.8–1232.0)	0.001
Interleukin-6, pg/mL	21.7 (7.3–53.9)	15.2 (3.9–40.0)	33.0 (12.0–68.5)	< 0.001
Interleukin-8, pg/mL	15.5 (9.4–26.6)	15.1 (9.2–26.0)	16.0 (9.6–28.2)	0.261
Interleukin-10, pg/mL	5.6 (5.00–9.4)	5.0 (5.0–8.5)	6.6 (5.0–10.2)	0.12
Tumor necrosis factor alpha, pg/mL	9.1 (7.2–12.1)	8.7 (6.8–11.6)	9.7 (7.6–13.0)	0.093

Data are expressed as median (IQR) or *n* (%)

### Association of variables with disease severity using univariate and multivariate logistic regression analysis

Odds ratio (OR) for the association of variables with COVID-19 severity was shown in Table 4. In the univariate logistic regression analysis, age (> 65 vs. ≤ 65 years), shortness of breath (yes vs. no), white blood cell count (> 10 vs. ≤ 10 × 10^9^/L), neutrophil count (> 7 vs. ≤ 7 × 10^9^/L), lymphocyte count (< 0.6 vs. ≥ 0.6 × 10^9^/L), platelet count (< 100 vs. ≥ 100 × 10^9^/L), albumin (< 30 vs. ≥ 30 g/L), blood bicarbonate ions (< 20 vs. ≥ 20 mmol/L), blood urea nitrogen (> 6 vs. ≤ 6 mmol/L), blood creatinine (> 100 vs. ≤ 100 μmol/L), LDH (> 400 vs. ≤ 400 U/L), hsCRP (> 90 vs. ≤ 90 mg/L), PCT (> 0.2 vs. ≤ 0.2 ng/L), Fer (> 1000 vs. ≤ 1000 μg/L), sIL-2R (> 1200 vs. ≤ 1200 U/mL), IL-6 (> 50 vs. ≤ 50 pg/mL), IL-8 (> 25 vs. ≤ 25 pg/mL), IL-10 (> 10 vs. ≤ 10 pg/mL), TNF-α (> 10 vs. ≤ 10 pg/mL), and hsCRP/L (> 120 vs. ≤ 120 × 10^−9^ mg) were associated with severity of COVID-19. By multivariate logistic regression analysis, we found that older age (OR 1.81 95% confidence interval (CI) 1.08–3.03), increased neutrophil count (OR 2.31, 95% CI 1.12–4.77), decreased platelet count (OR 2.09, 95% CI 1.15–3.79), elevated IL-6 (OR 2.02, 95% CI 1.11–4.03), and LDH (OR 5.45, 95% CI 2.42–12.26) were independently significant factors associating with COVID-19 severity.

**Table 4:** Factors associated with severity of COVID-19

	Univariate logistic regression			Multivariate logistic regression		
	OR	95% CI	*P* value	OR	95% CI	*P* value
Age >65 years	1.81	1.18–2.78	0.006	1.81	1.08–3.03	0.024
Males	1.36	0.89–2.08	0.152	0.69	0.41–1.16	0.162
Shortness of breath	1.92	1.25–2.94	0.003	1.48	0.91–2.42	0.115
**Laboratory findings**						
White blood cell count > 10 × 10^^^9/L	7.53	3.87–14.65	< 0.001	–	–	–
Neutrophil count > 7 × 10^^^9/L	7.60	4.21–13.74	< 0.001	2.31	1.12–4.77	0.024
Lymphocyte count < 0.6 × 10^^^9/L	3.55	2.11–5.99	< 0.001	1.23	0.66–2.29	0.522
Hemoglobin > 110 g/L	1.29	0.65–2.54	0.468	–	–	–
Platelet count < 100 × 10^^^9/L	2.38	1.45–3.93	0.001	2.09	1.15–3.79	0.015
Albumin < 30 g/L	4.11	2.36–7.16	< 0.001	1.02	0.49–2.12	0.959
Blood bicarbonate ions < 20 mmol/L	2.89	1.69–4.92	< 0.001	1.27	0.67–2.38	0.466
Blood urea nitrogen > 6 mmol/L	5.81	3.41–9.89	< 0.001	1.58	0.79–3.14	0.194
Blood creatinine > 100 μmol/L	2.84	1.66–4.85	< 0.001	1.14	0.56–2.31	0.717
Lactate dehydrogenase > 400 U/L	16.57	8.80–31.20	< 0.001	5.45	2.42–12.26	< 0.001
High-sensitivity C-reactive protein > 90 mg/L	4.83	2.84–8.21	< 0.001	1.21	0.58–2.50	0.615
Procalcitonin > 0.2 ng/mL	6.29	3.64–10.85	< 0.001	1.45	0.67–3.17	0.347
Ferritin > 1000 μg/L	4.06	2.42–6.82	< 0.001	1.06	0.52–2.13	0.876
Soluble interlukin-2 receptor > 1200 U/mL	5.20	3.06–8.86	< 0.001	1.60	0.78–3.30	0.198
Interlukin-6 > 50 pg/mL	4.88	2.86–8.33	< 0.001	2.02	1.11–4.03	0.045
Interlukin-8 > 25 pg/mL	2.65	1.61–4.36	< 0.001	1.10	0.61–2.00	0.749
Interlukin-10 > 10 pg/mL	2.77	1.70–4.52	< 0.001	1.30	0.72–2.35	0.383
Tumor necrosis factor alpha > 10 pg/mL	1.96	1.20–3.20	0.007	0.71	0.37–1.36	0.308
hsCRP/lymphocyte > 120 × 10^^^9 mg	7.14	4.10–12.44	< 0.001	–	–	–

## Discussion

In response to pathogens, host immune cells exhibit different reactions against the various infectious agents. Virus-cell interactions generate a diverse set of immune mediators against the invading virus [22, 23]. Although an effective immune response is essential to control and eliminate viral infection, an exaggerated or prolonged response could result in immunopathogenesis. Excessive production of inflammatory mediators is involved in the immunopathology and development of organ dysfunction [24, 25]. SARS-CoV and MERS-CoV infections predominantly affect lower airways and cause severe and sometime fatal pneumonia which is often characterized with massive infiltration of inflammatory cells and copious amounts of inflammatory mediators. Extrapulmonary organ dysfunction was also involved in those two CoV infections [26, 27]. SARS-CoV-2 infection resulted in multiple organ injury accompanied by high levels of serum inflammatory mediators, indicating that COVID-19 was not just lung disease, but rather a systemic inflammatory illness [3, 10, 28]. Longitudinal analysis of correlation of serum inflammatory parameters with different disease severity and outcomes may extend our understanding of the role of the host immune system in the pathogenesis and disease progression of COVID-19.

In this present study, the serum levels of inflammatory parameters in COVID-19 patients were analyzed and demonstrated that SARS-CoV-2 infection elicited a markedly elevated production of serum inflammatory parameters in severe and critically ill COVID-19 patients. The concentrations of pro-inflammatory cytokines, LDH, hsCRP, and hsCRP/L were gradually declined in moderate and severe patients as well as survivors after medical intervention, whereas they were sustained at high levels throughout the disease course in both critically ill patients and deceased cases.

Accumulating evidence has shown that several cytokines and inflammatory parameters were markedly elevated in severe patients with COVID-19 or those admitted to the ICU [3, 9, 20, 29]. Our previous preliminary study of 21 patients with COVID-19 exhibited that levels of sIL-2R, IL-10, TNF-α, hsCRP, Fer, and LDH were higher in the severe group than in the moderate group [19]. Consistent with those findings, concentrations of sIL-2R, IL-6, IL-8, IL-10, TNF-α, hsCRP, Fer, PCT, and LDH on admission were elevated significantly in critically ill patients than moderate and severe cases in the present study. Zhou previously reported that IL-6 was elevated with illness deterioration [8]. It is worth noting that the serum concentrations of inflammatory parameters in critically ill patients were markedly higher on admission, suggesting that vigilant monitoring and early intervention aiming to control overactive inflammation may be useful to prevent the further deterioration of COVID-19. The measurement of systemic inflammatory parameters on admission is important in determining the magnitude of the immune response and disease severity. Moreover, the monitoring the serial changes of these indicators during disease course may be of more value in clinical practice. At present, the information about correlation of longitudinal changes of inflammatory parameters with disease severity in COVID-19 patients is scarce. In this study, moderate and severe cases as well as survivors exhibited gradual decrease in concentrations of pro-inflammatory cytokines, hsCRP, and hsCRP/L throughout the disease course after receiving medical treatment, mainly including oxygen therapy, supportive therapy, and empirical antimicrobial therapy, whereas in critically ill patients and deceased cases, these markers sustained at high levels. The levels of serum cytokines, LDH, hsCRP, and hsCRP/L in survivors were significantly lower than those of deceased patients during the course of hospitalization. Taken together, sustained high levels of cytokines, LDH, hsCRP, and hsCRP/L may be associated with severe illness and poor prognosis.

IL-6, IL-8, and TNF-α are widely recognized as important potent initiators of inflammatory responses. Previous studies have shown that IL-6, IL-8, and TNF-α may promote inflammation by recruiting immune cells to the lung, which may directly contribute to the pathogenesis of ARDS [30]. Likewise, remarkably elevated serum pro-inflammatory cytokines were also found in SARS and MERS patients in severe condition compared to mild and moderate cases [18, 31–35]. Similar to SARS-CoV and MERS-CoV infection, high plasma pro-inflammatory cytokines (IL-6, IL-8 and TNF-α) were observed in severe and critically ill patients as well as deceased cases, suggesting a crucial role of exuberant inflammatory responses in SARS-CoV-2 infection pathogenesis. Excessive production of pro-inflammatory mediators released by activated immune cells and infected cells may be involved in immunopathology and the development of organ dysfunction.

The pro-inflammatory response is regulated by the anti-inflammatory components of the immune system. IL-10 with potent anti-inflammatory properties exerts suppressive effects on the production of several pro-inflammatory cytokines during lung injury [36, 37]. In patients with ARDS, higher concentrations of IL-10 are associated with better survival [38]. However, IL-10 levels were significant higher in severe patients with MERS than in mild cases and were positively correlated with mortality [35]. On the contrary, severe SARS patients had lower levels of IL-10 [39]. Similar to that in MERS-CoV infection, we found that IL-10 level was continuously elevated in critically ill patients and deceased cases with COVID-19, while IL-10 concentration transiently increased during hospitalization in severe cases and survivors and then fell to lowest level before discharge. Therefore, the transient increase of IL-10 level may reflect a compensatory anti-inflammatory or counter-regulatory reaction in response to a heightened level of pro-inflammatory cytokines, and sustained elevation of IL-10 is probably correlated with the poor prognosis. The differential alteration of IL-10 observed in SARS-CoV, MERS-CoV, and SARS-CoV-2 infection suggested that its anti-inflammatory regulation might differ among the three diseases.

Serum sIL-2R is considered as an activation marker of T cells [40, 41]. Raised concentrations of sIL-2R have been demonstrated in autoimmune disease and lymphoid malignancies in which enhanced T cell activity is centrally involved [42, 43]. The concentrations of serum sIL-2R were markedly higher in patients with subsequent acute lung injury (ALI) than those without [44]. Our data suggested that adaptive immune response might be over-reactive in severe and critically ill patients and deceased cases with COVID-19. Moreover, increasing serum sIL-2R levels may precede T cell-driven fibrotic responses [45]. Further investigation is required to determine the correlation between serum sIL-2R concentrations and pulmonary fibrosis after SARS-CoV-2 infection.

At present, no drugs have been proven to be effective against SARS-CoV-2 infection. Two adjunctive therapies that warrant special mention are corticosteroids and immunomodulatory or anti-cytokine therapy. A randomized, controlled trial reported that dexamethasone reduced 28-day mortality among severe or critically ill patients receiving invasive mechanical ventilation or oxygen at randomization [46]. A multicenter, single-blind, randomized controlled trial showed that ruxolitinib (JAK1/2 inhibitor) recipients with COVID-19 had a numerically faster clinical improvement [47]. Our results showed that IL-6 was predictive of disease severity, which was consistent with previous reports [48, 49]. Tocilizumab, a monoclonal antibody against IL-6, emerged as an alternative treatment for COVID-19 patients with a risk of cytokine storms recently [50]. Given the exuberant inflammatory response may be one of the hallmarks of severe COVID-19, therapeutic strategies to control overactive inflammation might be a promising approach for severe COVID-19; however, the optimal timing and dosing warrants further exploration.

There had been more discussion regarding the possible sex differences in the incidence and severity of the various infectious diseases. At present, sex-disaggregated data for COVID-19 show equal numbers of cases between males and females; however, there seem to be gender differences in vulnerability and mortality to SARS-CoV-2 infection [51]. Studies reported that more males than females died, possibly owing to sex-based immunological or gender differences, such as prevalence of smoking [52, 53]. It has been recognized that biological sex affects innate and adaptive immune responses to antigens [54, 55]. However, knowledge of gendered effect and its association with immune response of COVID-19 was scarce. Previous reports showed that hsCRP, Fer, LDH, and PCT levels varied between male and female patients [56]. In the present study, in addition to hsCRP, Fer, LDH, and PCT, sIL-2R and IL-6 levels were markedly higher in the serum of male patients compared with those of female cases, which implied that SARS-CoV-2 infection appears to elicit a sex-based differential immune response. Such sex-based immunological differences in COVID-19 might be partially attributed to sex hormone [57, 58], and the underlying mechanisms warrant further investigation.

Our study has some limitations. Firstly, we did not measure inflammatory mediators in bronchoalveolar lavage fluid. The autopsy of COVID-19 pneumonia indicated that SARS-CoV-2 infection caused an inflammatory cell infiltration in the lung tissue [13]. Since circulating inflammatory parameters concentrations may not exactly reflect the levels in injured lung tissue in some patients [59], it would be ideal to measure both local lung and systemic inflammatory parameters. Further studies are warranted to investigate the correlation between the local lung and systemic inflammatory parameters. Secondly, data on longitudinal changes of PCT and Fer are lacking; thus, we did not present the temporal changes of these acute-phase proteins.

## Conclusion

Collectively, the SARS-Cov-2 infection may result in hyper-reaction of the immune systems accompanied by elevated serum levels of inflammatory parameters, which may be associated with disease severity and outcomes. Moreover, SARS-CoV-2 infection appears to elicit a sex-based differential immune response. Evaluating the longitudinal changes of cytokines, LDH, hsCRP, and hsCRP/L levels might provide an effective way to evaluate the severity of disease and predict outcomes.

## Supplementary information

**Additional file 1: Table S1.** Signs, symptoms and treatment of patients infected with SARS-CoV-2. **Table S2.** Clinical characteristics, baseline laboratory findings and treatment of patients with longitudinal changes of inflammatory parameters.

**Additional file 2: Figure S1.** Laboratory findings of COVID-19 patients with different disease severity at three time points. **Figure S2.** Laboratory findings in survivors and deceased patients with COVID-19 at three time points.

## Data Availability

The datasets used in the present study are available from the first authors and corresponding authors on reasonable request.

## Abbreviations

COVID-19: Coronavirus disease 2019
SARS: Severe acute respiratory syndrome
MERS: Middle East respiratory syndrome
SARS-CoV: Severe acute respiratory syndrome coronavirus
SARS-CoV-2: Severe acute respiratory syndrome coronavirus 2
ARDS: Acute respiratory distress syndrome
ALI: Acute lung injury
ICU: Intensive care unit
hsCRP: High-sensitivity C-reactive protein
hsCRP/L: hsCRP to lymphocyte count ratio
sIL-2R: Soluble interleukin-2 receptor
IL-6: Interleukin-6
IL-8: Interleukin-8
IL-10: Interleukin-10
TNF-α: Tumor necrosis factor α
Fer: Ferritin
PCT: Procalcitonin
LDH: Lactate dehydrogenase

## References

[1] Drosten C, Gunther S, Preiser W, van der Werf S, Brodt HR, Becker S, Rabenau H, Panning M, Kolesnikova L, Fouchier RA (2003). Identification of a novel coronavirus in patients with severe acute respiratory syndrome. N Engl J Med.

[2] de Groot RJ, Baker SC, Baric RS, Brown CS, Drosten C, Enjuanes L, Fouchier RA, Galiano M, Gorbalenya AE, Memish ZA (2013). Middle East respiratory syndrome coronavirus (MERS-CoV): announcement of the Coronavirus Study Group. J Virol.

[3] Huang C, Wang Y, Li X, Ren L, Zhao J, Hu Y, Zhang L, Fan G, Xu J, Gu X (2020). Clinical features of patients infected with 2019 novel coronavirus in Wuhan, China. Lancet.

[4] Zhu N, Zhang D, Wang W, Li X, Yang B, Song J, Zhao X, Huang B, Shi W, Lu R (2020). A novel coronavirus from patients with pneumonia in China, 2019. N Engl J Med.

[5] Coronavirus disease (COVID-19) Situation Dashboard. 2020. https://www.who.int/emergencies/diseases/novel-coronavirus-2019. Accessed 9 July 2020.

[6] Bai Y, Yao L, Wei T, Tian F, Jin DY, Chen L, Wang M. Presumed asymptomatic carrier transmission of COVID-19. JAMA. 2020;323(14):1406–7. 10.1001/jama.2020.2565 PMC7042844 32083643

[7] Hu Z, Song C, Xu C, Jin G, Chen Y, Xu X, Ma H, Chen W, Lin Y, Zheng Y, et al. Clinical characteristics of 24 asymptomatic infections with COVID-19 screened among close contacts in Nanjing, China. Sci China Life Sci. 2020;63(5):706–11. 10.1007/s11427-020-1661-4 PMC7088568 32146694

[8] Zhou F, Yu T, Du R, Fan G, Liu Y, Liu Z, Xiang J, Wang Y, Song B, Gu X, et al. Clinical course and risk factors for mortality of adult inpatients with COVID-19 in Wuhan, China: a retrospective cohort study. Lancet. 2020;395(10229):1054–62. 10.1016/S0140-6736(20)30566-3 PMC7270627 32171076

[9] Wang D, Hu B, Hu C, Zhu F, Liu X, Zhang J, Wang B, Xiang H, Cheng Z, Xiong Y, et al. Clinical characteristics of 138 hospitalized patients with 2019 novel coronavirus-infected pneumonia in Wuhan, China. JAMA 2020;323(11):1061–9. 10.1001/jama.2020.1585 PMC7042881 32031570

[10] Chen T, Wu D, Chen H, Yan W, Yang D, Chen G, Ma K, Xu D, Yu H, Wang H (2020). Clinical characteristics of 113 deceased patients with coronavirus disease 2019: retrospective study. BMJ.

[11] Cao X (2020). COVID-19: immunopathology and its implications for therapy. Nat Rev Immunol.

[12] Tan L, Wang Q, Zhang D, Ding J, Huang Q, Tang YQ, Wang Q, Miao H (2020). Lymphopenia predicts disease severity of COVID-19: a descriptive and predictive study. Signal Transduct Target The.

[13] Xu Z, Shi L, Wang Y, Zhang J, Huang L, Zhang C, Liu S, Zhao P, Liu H, Zhu L, et al. Pathological findings of COVID-19 associated with acute respiratory distress syndrome. Lancet Respir Med. 2020;8(4):420–2. 10.1016/S2213-2600(20)30076-X PMC7164771 32085846

[14] Yang X, Yu Y, Xu J, Shu H, Xia JA, Liu H, Wu Y, Zhang L, Yu Z, Fang M, et al. Clinical course and outcomes of critically ill patients with SARS-CoV-2 pneumonia in Wuhan, China: a single-centered, retrospective, observational study. Lancet Respir Med. 2020;8(5):475–81. 10.1016/S2213-2600(20)30079-5 PMC7102538 32105632

[15] Cui Y, Tian M, Huang D, Wang X, Huang Y, Fan L, Wang L, Chen Y, Liu W, Zhang K, et al. A 55-day-old female infant infected with COVID 19: presenting with pneumonia, liver injury, and heart damage. J Infect Dis. 2020;221(11):1775–81. 10.1093/infdis/jiaa113 PMC7184483 32179908

[16] Lau SKP, Lau CCY, Chan KH, Li CPY, Chen H, Jin DY, Chan JFW, Woo PCY, Yuen KY (2013). Delayed induction of proinflammatory cytokines and suppression of innate antiviral response by the novel Middle East respiratory syndrome coronavirus: implications for pathogenesis and treatment. J Gen Virol.

[17] Mahallawi WH, Zhang Q, Makhdoum HM, Suliman BA, Khabour OF (2018). MERS-CoV infection in humans is associated with a pro-inflammatory Th1 and Th17 cytokine profile. Cytokine.

[18] Channappanavar R, Perlman S (2017). Pathogenic human coronavirus infections: causes and consequences of cytokine storm and immunopathology. Semin Immunopathol.

[19] Chen G, Wu D, Guo W, Cao Y, Huang D, Wang H, Wang T, Zhang X, Chen H, Yu H, et al. Clinical and immunologic features in severe and moderate coronavirus disease 2019. J Clin Invest. 2020;130(5):2620–9. 10.1172/JCI137244 PMC7190990 32217835

[20] Qin C, Zhou L, Hu Z, Zhang S, Yang S, Tao Y, Xie C, Ma K, Shang K, Wang W, et al. Dysregulation of immune response in patients with COVID-19 in Wuhan, China. Clin Infect Dis. 2020;71(15):762–8. 10.1093/cid/ciaa248 PMC7108125 32161940

[21] The notice of launching guideline on diagnosis and treatment of the novel coronavirus pneumonia (NCP).6th edition http://www.nhc.gov.cn/yzygj/s7653p/202002/8334a83 -26dd94d329df351d7da8aefc2/files/b218cfeb1bc54639af227f922bf6b817.Accessed 27 Mar 2020. National Health Commission of the People’s Republic of China.

[22] Iwasaki A, Pillai PS (2014). Innate immunity to influenza virus infection. Nat Rev Immunol.

[23] Takeuchi O, Akira S (2009). Innate immunity to virus infection. Immunol Rev.

[24] Wang H, Ma S (2008). The cytokine storm and factors determining the sequence and severity of organ dysfunction in multiple organ dysfunction syndrome. Am J Emerg Med.

[25] Huang KJ, Su IJ, Theron M, Wu YC, Lai SK, Liu CC, Lei HY (2005). An interferon-gamma-related cytokine storm in SARS patients. J Med Virol.

[26] Peiris JS, Chu CM, Cheng VC, Chan KS, Hung IF, Poon LL, Law KI, Tang BS, Hon TY, Chan CS (2003). Clinical progression and viral load in a community outbreak of coronavirus-associated SARS pneumonia: a prospective study. Lancet.

[27] Oboho IK, Tomczyk SM, Al-Asmari AM, Banjar AA, Al-Mugti H, Aloraini MS, Alkhaldi KZ, Almohammadi EL, Alraddadi BM, Gerber SI (2015). 2014 MERS-CoV outbreak in Jeddah--a link to health care facilities. N Engl J Med.

[28] Wang T, Du Z, Zhu F, Cao Z, An Y, Gao Y, Jiang B (2020). Comorbidities and multi-organ injuries in the treatment of COVID-19. Lancet.

[29] Gao Y, Li T, Han M, Li X, Wu D, Xu Y, Zhu Y, Liu Y, Wang X, Wang L. Diagnostic utility of clinical laboratory data determinations for patients with the severe COVID-19. J Med Virol. 2020;92(7):791–6. 10.1002/jmv.25770 PMC7228247 32181911

[30] Goodman RB, Strieter RM, Martin DP, Steinberg KP, Milberg JA, Maunder RJ, Kunkel SL, Walz A, Hudson LD, Martin TR (1996). Inflammatory cytokines in patients with persistence of the acute respiratory distress syndrome. Am J Respir Crit Care Med.

[31] Wong CK, Lam CW, Wu AK, Ip WK, Lee NL, Chan IH, Lit LC, Hui DS, Chan MH, Chung SS (2004). Plasma inflammatory cytokines and chemokines in severe acute respiratory syndrome. Clin Exp Immunol.

[32] Zhang Y, Li J, Zhan Y, Wu L, Yu X, Zhang W, Ye L, Xu S, Sun R, Wang Y (2004). Analysis of serum cytokines in patients with severe acute respiratory syndrome. Infect Immun.

[33] Sheng WH, Chiang BL, Chang SC, Ho HN, Wang JT, Chen YC, Hsiao CH, Hseuh PR, Chie WC, Yang PC (2005). Clinical manifestations and inflammatory cytokine responses in patients with severe acute respiratory syndrome. J Formosan Med Assoc.

[34] Kim ES, Choe PG, Park WB, Oh HS, Kim EJ, Nam EY, Na SH, Kim M, Song KH, Bang JH (2016). Clinical progression and cytokine profiles of Middle East respiratory syndrome coronavirus infection. J Korean Med Sci.

[35] Min CK, Cheon S, Ha NY, Sohn KM, Kim Y, Aigerim A, Shin HM, Choi JY, Inn KS, Kim JH (2016). Comparative and kinetic analysis of viral shedding and immunological responses in MERS patients representing a broad spectrum of disease severity. Sci Rep.

[36] Goodman RB, Pugin J, Lee JS, Matthay MA (2003). Cytokine-mediated inflammation in acute lung injury. Cytokine Growth Factor Rev.

[37] Antunes G, Evans SA, Lordan JL, Frew AJ (2002). Systemic cytokine levels in community-acquired pneumonia and their association with disease severity. Eur Respir J.

[38] Miyaoka K, Iwase M, Suzuki R, Kondo G, Watanabe H, Ito D, Nagumo M. Clinical evaluation of circulating interleukin-6 and interleukin-10 levels after surgery-induced inflammation. J Surg Res. 2005;125(2):144–50. 10.1016/j.jss.2004.12.001 15854666

[39] Chien JY, Hsueh PR, Cheng WC, Yu CJ, Yang PC (2006). Temporal changes in cytokine/chemokine profiles and pulmonary involvement in severe acute respiratory syndrome. Respirology.

[40] Turka LA, Walsh PT (2008). IL-2 signaling and CD4+ CD25+ Foxp3+ regulatory T cells. Front Biosci.

[41] Lin M, Park S, Hayden A, Giustini D, Trinkaus M, Pudek M, Mattman A, Schneider M, Chen LYC (2017). Clinical utility of soluble interleukin-2 receptor in hemophagocytic syndromes: a systematic scoping review. Ann Hematol.

[42] Kitagawa J, Hara T, Tsurumi H, Goto N, Kanemura N, Yoshikawa T, Kasahara S, Yamada T, Sawada M, Takahashi T (2009). Serum-soluble interleukin-2 receptor (sIL-2R) is an extremely strong prognostic factor for patients with peripheral T-cell lymphoma, unspecified (PTCL-U). J Cancer Res Clin Oncol.

[43] Karim AF, Eurelings LEM, Bansie RD, van Hagen PM, van Laar JAM, Dik WA (2018). Soluble interleukin-2 receptor: a potential marker for monitoring disease activity in IgG4-related disease. Mediat Inflamm.

[44] Takala A, Jousela I, Takkunen O, Kautiainen H, Jansson SE, Orpana A, Karonen SL, Repo H (2002). A prospective study of inflammation markers in patients at risk of indirect acute lung injury. Shock.

[45] Betjes MG, Habib MS, Struijk DG, Lopes Barreto D, Korte MR, Abrahams AC, Nagtzaam NM, Clahsen-van Groningen MC, Dik WA, Litjens NH. Encapsulating peritoneal sclerosis is associated with T-cell activation. Nephrol Dial Transplant. 2015;30(9):1568–76. 10.1093/ndt/gfv092 25934991

[46] Mafham PHWSLJEM. Effect of Dexamethasone in Hospitalized Patients with COVID-19: Preliminary Report. medRxiv. 2020;06.22.20137273.

[47] Cao Y, Wei J, Zou L, Jiang T, Wang G, Chen L, Huang L, Meng F, Huang L, Wang N, et al. Ruxolitinib in treatment of severe coronavirus disease 2019 (COVID-19): a multicenter, single-blind, randomized controlled trial. J Allergy Clin Immunol. 2020;146(1):137–46 e133. 10.1016/j.jaci.2020.05.019 PMC7250105 32470486

[48] Han H, Ma Q, Li C, Liu R, Zhao L, Wang W, Zhang P, Liu X, Gao G, Liu F, et al. Profiling serum cytokines in COVID-19 patients reveals IL-6 and IL-10 are disease severity predictors. Emerg Microbes Infect. 2020;9(1):1123–30. 10.1080/22221751.2020.1770129 PMC7473317 32475230

[49] Del Valle DM, Kim-Schulze S, Hsin-Hui H, Beckmann ND, Nirenberg S, Wang B, Lavin Y, Swartz T, Madduri D, Stock A, et al. An inflammatory cytokine signature helps predict COVID-19 severity and death. medRxiv. 2020;2020.05.28.20115758. 10.1038/s41591-020-1051-9 PMC7869028 32839624

[50] Luo P, Liu Y, Qiu L, Liu X, Liu D, Li J. Tocilizumab treatment in COVID-19: a single center experience. J Med Virol. 2020;92(7):814–8. 10.1002/jmv.25801 PMC7262125 32253759

[51] Wenham C, Smith J, Morgan R, Gender, Group C-W. COVID-19: the gendered impacts of the outbreak. Lancet. 2020;395(10227):846–8. 10.1016/S0140-6736(20)30526-2 PMC7124625 32151325

[52] Chen N, Zhou M, Dong X, Qu J, Gong F, Han Y, Qiu Y, Wang J, Liu Y, Wei Y, et al. Epidemiological and clinical characteristics of 99 cases of 2019 novel coronavirus pneumonia in Wuhan, China: a descriptive study. Lancet. 2020;395(10223):507–13. 10.1016/S0140-6736(20)30211-7 PMC7135076 32007143

[53] Liu S, Zhang M, Yang L, Li Y, Wang L, Huang Z, Wang L, Chen Z, Zhou M. Prevalence and patterns of tobacco smoking among Chinese adult men and women: findings of the 2010 national smoking survey. J Epidemiol Community Health. 2017;71(2):154–61. 10.1136/jech-2016-207805 PMC5284482 27660401

[54] Klein SL, Flanagan KL. Sex differences in immune responses. Nat Rev Immunol. 2016;16(10):626–38. 10.1038/nri.2016.90 27546235

[55] Markle JG, Fish EN. SeXX matters in immunity. Trends Immunol. 2014;35(3):97–104. 10.1016/j.it.2013.10.006 24239225

[56] Meng Y, Wu P, Lu W, Liu K, Ma K, Huang L, Cai J, Zhang H, Qin Y, Sun H, et al. Sex-specific clinical characteristics and prognosis of coronavirus disease-19 infection in Wuhan, China: a retrospective study of 168 severe patients. PLoS Pathog. 2020;16(4):e1008520. 10.1371/journal.ppat.1008520 PMC7209966 32343745

[57] Gargaglioni LH, Marques DA. Let’s talk about sex in the context of COVID-19. J Appl Physiol. 2020;128(6):1533–8. 10.1152/japplphysiol.00335.2020 PMC7303729 32437244

[58] Kadel S, Kovats S. Sex hormones regulate innate immune cells and promote sex differences in respiratory virus infection. Front Immunol. 2018;9:1653. 10.3389/fimmu.2018.01653 PMC6062604 30079065

[59] Schutte H, Lohmeyer J, Rosseau S, Ziegler S, Siebert C, Kielisch H, Pralle H, Grimminger F, Morr H, Seeger W. Bronchoalveolar and systemic cytokine profiles in patients with ARDS, severe pneumonia and cardiogenic pulmonary oedema. Eur Respir J. 1996;9(9):1858–67. 10.1183/09031936.96.09091858 8880103

