# Supplementary material for: Longitudinal changes of inflammatory parameters and their correlation with disease severity and outcomes in patients with COVID-19 from Wuhan, China
Source: Crit Care. 2020 Aug 27;24:525. doi: 10.1186/s13054-020-03255-0 (PMC7450961; doi:10.1186/s13054-020-03255-0)
Supplement: Supplementary file 1 — Additional file 1: Table S1. Signs, symptoms and treatment of patients infected with SARS-CoV-2. Table S2. Clinical characteristics, baseline laboratory findings and treatment of patients with longitudinal changes of inflammatory parameters. [file 13054_2020_3255_MOESM1_ESM.docx]

| Table S1. Signs, symptoms and treatment of patients infected with SARS-CoV-2 | | | | | | | | | | | |
| --- | --- | --- | --- | --- | --- | --- | --- | --- | --- | --- | --- |
|  | | All patients (n=317) | | Moderate patients (n=93) | | | Severe patients (n=167) | | Critical patients (n=57) | |  |
| **Signs and symptoms** | |  | |  | | |  | |  | |  |
| Fever, n (%) | | 284（89.6%） | | 80(86.0%) | | | 154(92.2%) | | 50(87.7%) | |  |
| Cough, n (%) | | 240（75.7%） | | 66(71.0%) | | | 128(76.6%) | | 46(80.7%) | |  |
| Diarrhea, n (%) | | 68（21.5%） | | 20(21.5%) | | | 38(22.8%) | | 10(17.5%) | |  |
| Fatigue, n (%) | | 102(32.2%) | | 28(30.1%) | | | 48(28.7%) | | 26(45.6%) | |  |
| Shortness of breath, n (%) | | 150(47.3%) | | 34(36.6%) | | | 81(48.5%) | | 35(61.4%)^*^ | |  |
| Heart rate, per min | | 90(80-102) | | 90(80-102) | | | 89(80-100) | | 94(82-108) | |  |
| Respiratory rate, per min | | 20(20-22) | | 20(20-21) | | | 20(20-22) | | 22(20-28)^*#^ | |  |
| Systolic blood pressure, mmHg | | 128.0(118.0-143.0) | | 128.0(116.0-141.5) | | | 128.0(118.0-141.0) | | 130.0(120.0-145.0) | |  |
| Diastolic blood pressure, mmHg | | 80.0(80.0-89.0) | | 82.0(74.0-90.0) | | | 78.0(70.0-87.0) | | 82.0(73.5-92.0) | |  |
| **Treatment** | |  | |  | | |  | |  | |  |
| Antibiotics, n (%) | | 246(77.6%) | | 61(65.6%) | | | 130(77.8%)^*^ | | 55(96.5%)^*#^ | |  |
| Antivirus, n (%) | | 288(90.8%) | | 85(91.4%) | | | 153(91.6%) | | 50(87.7%) | |  |
| Glucocorticoid therapy, n (%) | | 155(48.9%) | | 27(29.0%) | | | 82(49.1%)^*^ | | 46(80.7%)^*#^ | |  |
| Intravenous immunoglobulin, n (%) | | 91(28.7%) | | 20(21.5%) | | | 41(24.6%) | | 30(52.6%)^*#^ | |  |
| High oxygen flow, n (%) | | 15(4.7%) | | 0 | | | 3(1.8%) | | 12(21.1%)^*#^ | |  |
| Noninvasive ventilation, n (%) | | 26(8.2%) | | 0 | | | 4(2.4%) | | 22(38.6%)^*#^ | |  |
| Invasive mechanical ventilation, n (%) | | 28(8.8%) | | 0 | | | 0 | | 28(49.1%)^*#^ | |  |
| Data are expressed as median (IQR) or n (%). ^*^P <0.05 represents significant differences between severe or critically ill group and moderate group, ^#^P <0.05 represents significant differences between moderate or critically ill group vs. severe group. | | | | | | | | | | |  |
| Table S2. Clinical characteristics, baseline laboratory findings and treatment of patients with longitudinal changes of inflammatory parameters | | | | | | | | | | | |
|  | All patients (n=68) | | Moderate patients (n=18) | | | Severe patients (n=29) | | Critical patients (n=21) | |  |  |
| **Characteristics** |  | |  | | |  | |  | |  |  |
| Age, yrs | 66.5(56.3-71.0) | | 64.0(52.3-69.0) | | | 67.0(60.5-71.0) | | 67.0(55.0-77.0) | |  |  |
| Males, n (%) | 38(55.9%) | | 7(38.9%) | | | 18(62.1%) | | 13(61.9%) | |  |  |
| comorbidity |  | |  | | |  | |  | |  |  |
| Chronic respiratory diseases, n (%) | 3(4.4%) | | 0 | | | 2(6.9%) | | 1(4.8%) | |  |  |
| Hypertension, n (%) | 26(38.2%) | | 5(27.8%) | | | 16(55.2%) | | 5(23.8%)^#^ | |  |  |
| Coronary artery disease, n (%) | 3(4.4%) | | 1(5.6%) | | | 2(6.9%) | | 0 | |  |  |
| Diabetes mellitus, n (%) | 12(17.6%) | | 2(11.1%) | | | 6(20.7%) | | 4(19.0%) | |  |  |
| Chronic kidney disease, n (%) | 1(1.5%) | | 1(5.5%) | | | 0 | | 0 | |  |  |
| Tumor, n(%) | 2(2.9%) | | 0 | | | 1(3.4%) | | 1(4.8%) | |  |  |
| **Signs and symptoms** |  | |  | | |  | |  | |  |  |
| Fever, n (%) | 61(89.7%) | | 17(94.4%) | | | 26(89.7%) | | 18(86.7%) | |  |  |
| Cough, n (%) | 53(77.9%) | | 16(88.9%) | | | 20(70.0%) | | 17(81.0%) | |  |  |
| Diarrhea, n (%) | 14(20.6%) | | 5(27.8%) | | | 5(17.2%) | | 4(19.1%) | |  |  |
| Fatigue, n (%) | 21(30.8%) | | 4(22.2%) | | | 8(27.6%) | | 9(42.9%) | |  |  |
| Shortness of breath, n (%) | 33(48.5%) | | 6(33.3%) | | | 15(51.7%) | | 12(57.1%) | |  |  |
| Heart rate, per min | 89.0(78.5-101.5) | | 90.0(78.0-99.8) | | | 88.0(83.0-101.5) | | 88.0(78.0-102.0) | |  |  |
| Respiratory rate, per min | 20.0(20.0-22.0) | | 20.0(20.0-21.3) | | | 20.0(20.0-22.0) | | 21.0(20.0-25.0) | |  |  |
| Systolic blood pressure, mmHg | 127.5(118.5-140.3) | | 122.5(114.8-133.3) | | | 129.0(120.0-145.5) | | 128.0(117.5-144.0) | |  |  |
| Diastolic blood pressure, mmHg | 80.0(73.3-89.0) | | 76.5(70.5-80.8) | | | 85.0(75.0-89.0) | | 85.0(72.0-97.5) | |  |  |
| **Laboratory findings** |  | |  | | |  | |  | |  |  |
| White blood cell count, ×10^9/L | 6.5(4.5-9.3) | | 4.6(3.3-6.4) | | | 6.7(4.7-9.4)^*^ | | 8.6(5.9-11.4)^*^ | |  |  |
| Neutrophil count, ×10^9/L | 4.9(3.1-7.8) | | 3.1(2.4-4.4) | | | 5.3(3.3-8.0)^*^ | | 7.0(4.9-10.1)^*^ | |  |  |
| Lymphocyte count, ×10^9/L | 0.8(0.7-1.1) | | 0.8(0.7-1.11) | | | 0.9(0.7-1.12) | | 0.7(0.5-0.9) | |  |  |
| Hemoglobin, g/L | 130.5(117.5-141.8) | | 129.0(111.0-141.3) | | | 124.0(116.0-142.0) | | 138.0(128.5-143.5) | |  |  |
| Platelet count, ×10^9/L | 229.5(168.8-293.3) | | 202.5(168.0-287.5) | | | 242.0(182.5-301.5) | | 223.0(148.0-304.0) | |  |  |
| Albumin, g/L | 32.0(29.2-34.0) | | 33.5(30.7-35.7) | | | 31.7(29.0-34.0) | | 30.7(28.7-33.6) | |  |  |
| Blood bicarbonate ions, mmol/L | 23.6(22.1-24.8) | | 23.9(22.5-24.7) | | | 23.8(22.4-25.0) | | 22.5(19.7-24.3) | |  |  |
| Blood urea nitrogen, mmol/L | 4.9(3.6-7.1) | | 3.7(3.1-4.3) | | | 4.7(3.8-6.8) | | 7.1(5.2-10.0)^*^ | |  |  |
| Blood creatinine, μmol/L | 71.0(61.5-87.0) | | 66.5(53.5-75.3) | | | 69.0(64.0-84.5) | | 78.0(64.5-97.0) | |  |  |
| hsCRP/ Lymphocyte, ×10^9 mg | 94.4(45.9-184.1) | | 62.8(28.0-109.7) | | | 87.0(40.1-151.7) | | 138.8(77.6-298.3)^*^ | |  |  |
| **Treatment** |  | |  | | |  | |  | |  |  |
| Antibiotics, n (%) | 59(86.8%) | | 15(83.3%) | | | 23(79.3%) | | 21(100.0%) | |  |  |
| Antivirus, n (%) | 63(92.6%) | | | | 16(88.9%) | 27(93.1%) | | 20(95.2%) | |  |  |
| Glucocorticoid therapy, n (%) | 41(60.3%) | | | | 5(27.8%) | 7(24.1%) | | 18(85.7%)^*#^ | |  |  |
| Intravenous immunoglobulin, n (%) | 26(38.2%) | | | | 5(27.8%) | 7(24.0%) | | 14(66.7%)^*#^ | |  |  |
| High oxygen flow, n (%) | 7(10.3%) | | | | 0 | 1(3.4%) | | 6(28.5%)^*#^ | |  |  |
| Noninvasive ventilation, n (%) | 10(14.7%) | | | | 0 | 3(10.3%) | | 7(33.3%)^*#^ | |  |  |
| Invasive mechanical ventilation, n (%) | 13(19.1%) | | | | 0 | 0 | | 13(61.9)^*#^ | |  |  |
| Data are expressed as median (IQR) or n (%). *P <0.05 represents significant differences between severe or critically ill group and moderate group, ^#^P <0.05 represents significant differences between moderate or critically ill group vs. severe group. | | | | | | | | | | | |
