# Supplementary material for: Longitudinal changes of inflammatory parameters and their correlation with disease severity and outcomes in patients with COVID-19 from Wuhan, China
Source: Crit Care. 2020 Aug 27;24:525. doi: 10.1186/s13054-020-03255-0 (PMC7450961; doi:10.1186/s13054-020-03255-0)
Supplement: Supplementary file 2 — Additional file 2: Figure S1. Laboratory findings of COVID-19 patients with different disease severity at three time points. Figure S2. Laboratory findings in survivors and deceased patients with COVID-19 at three time points. [file 13054_2020_3255_MOESM2_ESM.docx]

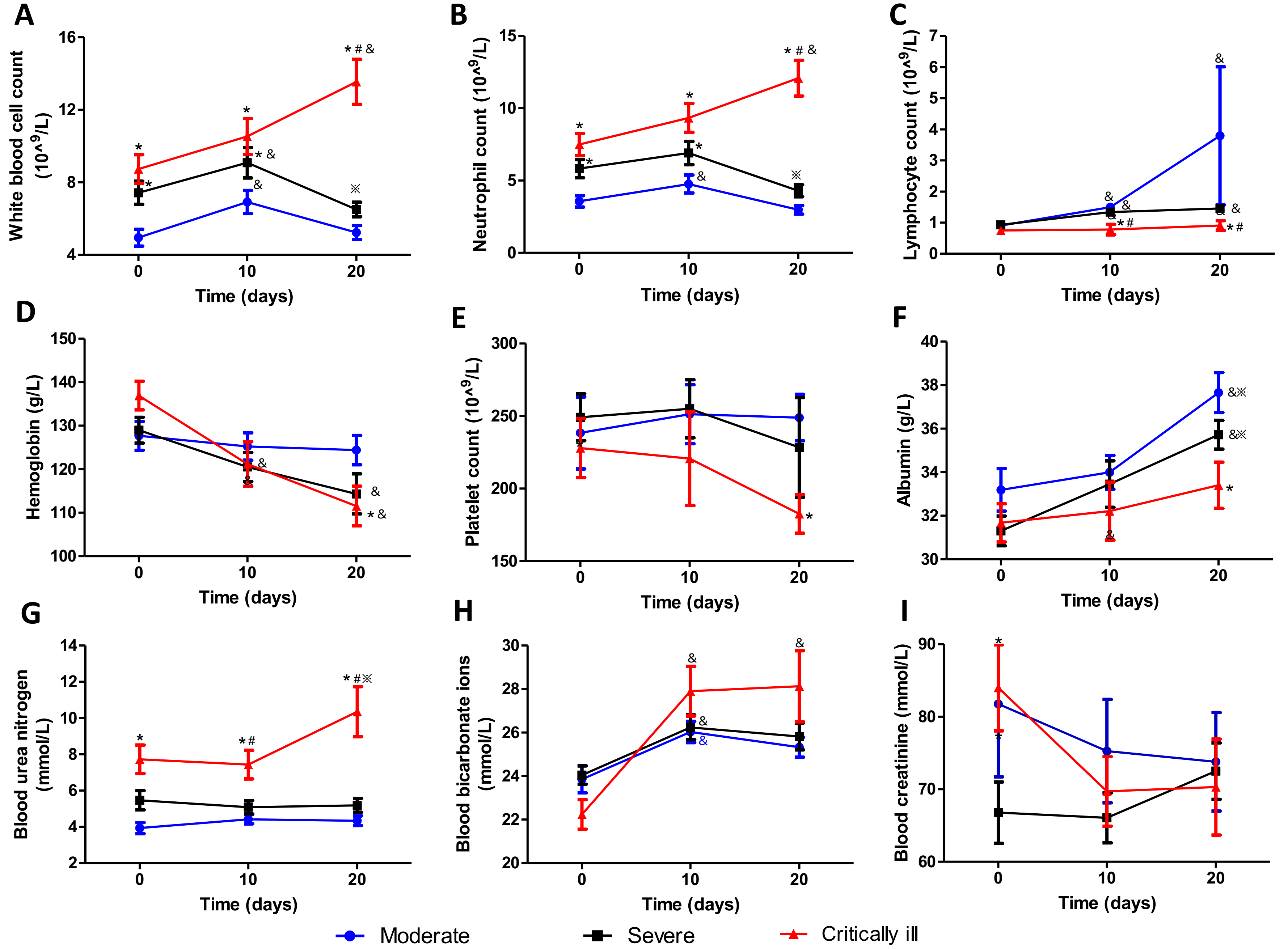


Figure S1. Laboratory findings of COVID-19 patients with different disease severity at three time points
The levels of white blood cell count (a), neutrophil count (b), lymphocyte count (c), hemoglobin (d), platelet count (e), albumin (f), blood bicarbonate ions (g), blood urea nitrogen (h) and blood creatinine(i) in moderate (blue circle, N=18), severe (black square, N=29) and critically ill (red triangle, N=21) patients at various time points were present. Data are expressed as mean±SEM. ^*^P <0.05 indicates difference between severe or critically ill patients versus the moderate patients, ^#^P <0.05 indicates difference between moderate or critically ill patients versus the severe patients, ^&^P <0.05 indicates difference between day 10 or 20 versus day 0, ^※^P <0.05 indicates difference between day 0 or 20 versus day 10.


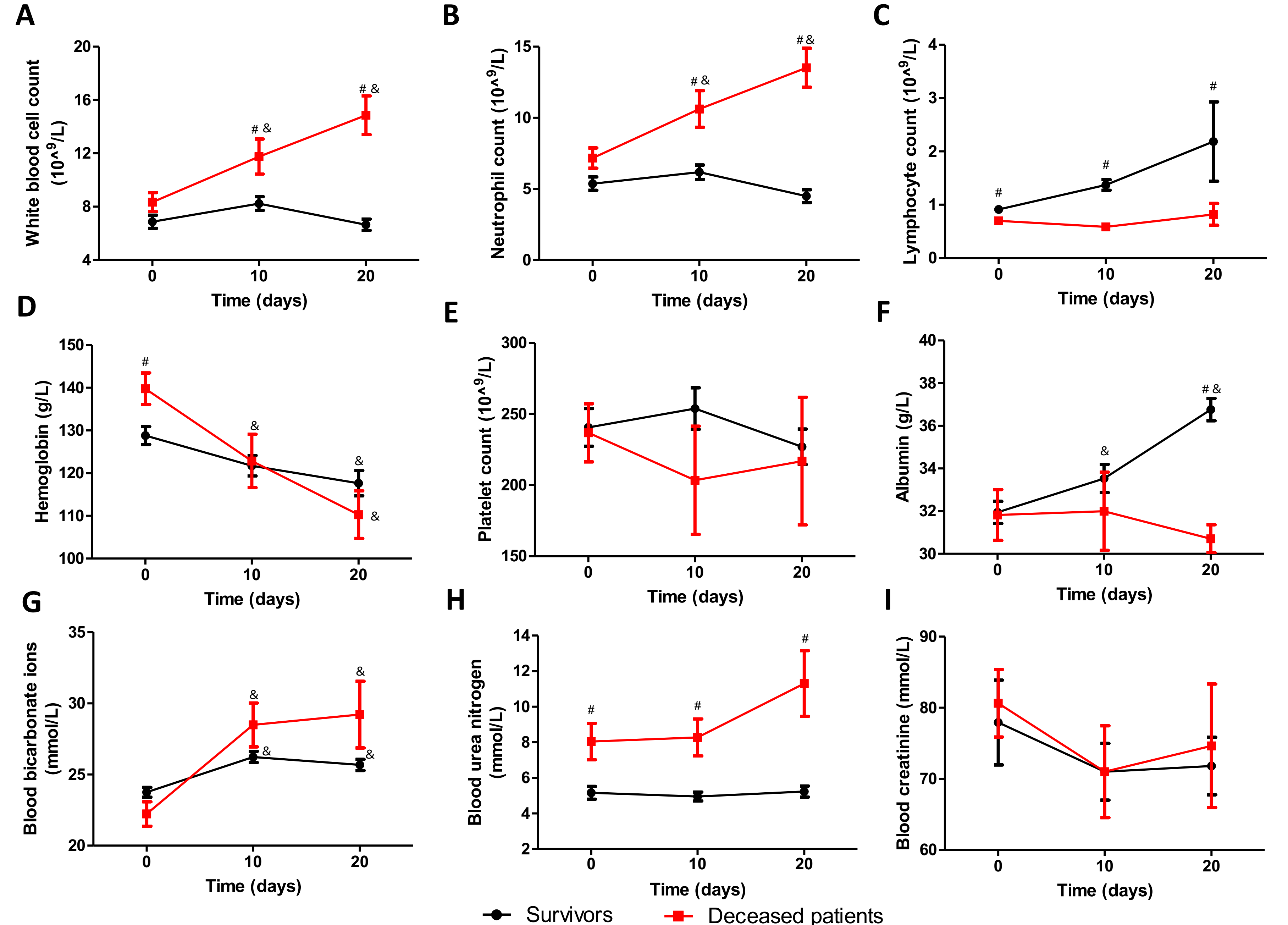


Figure S2. Laboratory findings in survivors and deceased patients with COVID-19 at three time points

The levels of white blood cell count (a), neutrophil count (b), lymphocyte count (c), hemoglobin (d), platelet count (e), albumin (f), blood bicarbonate ions (g), blood urea nitrogen (h) and blood creatinine(i) in survivors (black circle, N=54) and deceased patients (red square, N=14) were determined at various time points. Data are expressed as mean±SEM. ^#^P <0.05 indicates difference between survivors and deceased patients, ^&^P <0.05 indicates difference between day 10 or 20 versus day 0, ^※^P <0.05 indicates difference between day 0 or 20 versus day 10.
